# State-Dependent Network Connectivity Determines Gating in a K^+^ Channel

**DOI:** 10.1016/j.str.2014.04.018

**Published:** 2014-07-08

**Authors:** Murali K. Bollepalli, Philip W. Fowler, Markus Rapedius, Lijun Shang, Mark S.P. Sansom, Stephen J. Tucker, Thomas Baukrowitz

**Affiliations:** 1 Physiological Institute, Christian-Albrechts University, 24118 Kiel, Germany; 2 Department of Biochemistry, University of Oxford, Oxford OX1 3QU, UK; 3 Clarendon Laboratory, Department of Physics, University of Oxford Oxford, OX1 3PU, UK; 4 OXION Ion Channel Initiative, University of Oxford, Oxford OX1 3PT, UK

## Abstract

X-ray crystallography has provided tremendous insight into the different structural states of membrane proteins and, in particular, of ion channels. However, the molecular forces that determine the thermodynamic stability of a particular state are poorly understood. Here we analyze the different X-ray structures of an inwardly rectifying potassium channel (Kir1.1) in relation to functional data we obtained for over 190 mutants in Kir1.1. This mutagenic perturbation analysis uncovered an extensive, state-dependent network of physically interacting residues that stabilizes the pre-open and open states of the channel, but fragments upon channel closure. We demonstrate that this gating network is an important structural determinant of the thermodynamic stability of these different gating states and determines the impact of individual mutations on channel function. These results have important implications for our understanding of not only K^+^ channel gating but also the more general nature of conformational transitions that occur in other allosteric proteins.

## Highlights

• Functional validation of different crystallographic states of Kir channels
• Presence of a state-dependent gating network revealed by large-scale mutagenesis
• Biased effect of mutations on Kir channel gating due to open-state destabilization
• Long-range allosteric coupling mediated by a physically connected residue network

## Graphical Abstract

**Figure:**
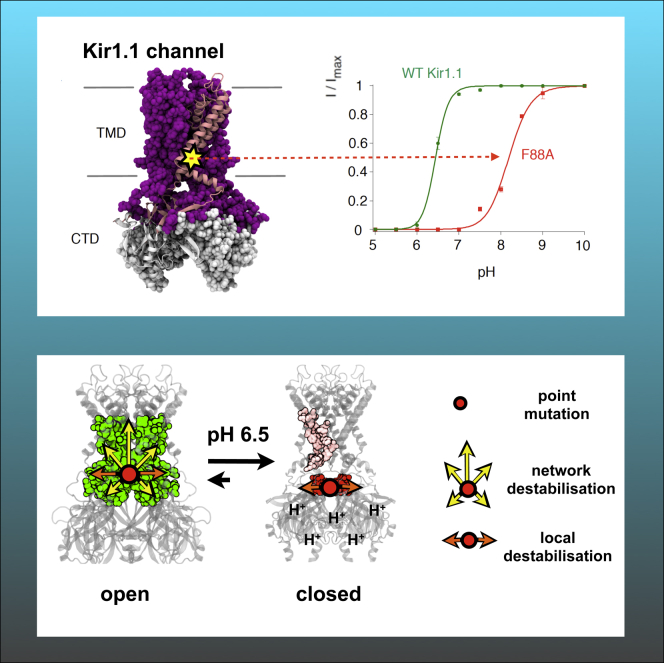

## Teaser

The molecular forces that determine the thermodynamic stability of ion channel gating states are poorly understood. Here, Bollepalli et al. show that an extensive state-dependent network of physically interacting residues determines the thermodynamic stability of the different gating states in Kir channels.

## Introduction

Ion channels play important and diverse roles in the control of cellular electrical excitability as well as many ion transport pathways. One of their most important properties is the ability to gate (i.e., to open and close) in response to a variety of physiological stimuli. Ion channel gating requires dynamic conformational changes, and our understanding of this process has been revolutionized over the past decade by advances in X-ray crystallography. These advances have revealed not only many individual channel structures but also examples of the same channel in several different conformational states. However, one of the major challenges in ion channel structural biology is understanding how these different crystallographic states relate to the functional properties of the channel (Alam and Jiang, 2009; Bavro et al., 2012; Cuello et al., 2010; Hansen et al., 2011; Prevost et al., 2012).

Inwardly rectifying (Kir) channels are an example where many different structural states have been determined, thus making them excellent candidates to probe the functional relationship between these different conformations (Bavro et al., 2012; Clarke et al., 2010; Hansen et al., 2011; Tao et al., 2009; Whorton and MacKinnon, 2011, 2013). In particular, the structures of Kir2.2, obtained both with and without a bound phosphoinositide (PIP_2_), suggest that pore opening is initiated by an upward movement of the cytoplasmic domain (CTD) that then engages with the slide helix and transmembrane-pore domain (TMD) to produce a “pre-open” PIP_2_-bound structure (Hansen et al., 2011; Tao et al., 2009). However, for the channel to become fully conductive, it is proposed that a further rotation of the transmembrane (TM) helices then occurs to open the helix bundle crossing (HBC) gate as observed in the crystal structures of PIP_2_-bound Kir3.2 and the putative open-state structure of the prokaryotic KirBac3.1 (Bavro et al., 2012; Whorton and MacKinnon, 2011, 2013). These different structural states therefore allow reconstruction of a possible gating pathway for the Kir channel, and we have chosen the pH-sensitive Kir1.1 (ROMK) channel to functionally probe this structural gating scheme.

Like many other Kir channels, Kir1.1 is inhibited by intracellular H^+^ but is activated by phosphoinositides such as PIP_2_. Functional studies suggest that both ligands control a similar gating mechanism at the HBC (Logothetis et al., 2007; Rapedius et al., 2006, 2007a, 2007b; Tucker and Baukrowitz, 2008; Zhang et al., 2006). However, studies of the kinetics of PIP_2_ and pH gating indicate that lowering the pH does not induce channel closure by simply promoting the unbinding of PIP_2_, but rather that pH gating occurs on a more rapid timescale with PIP_2_ still bound to the channel (Rapedius et al., 2007b). The precise mechanism of pH sensing in Kir channels remains elusive but is now thought to involve a number of titratable interactions that preferentially stabilize the open state of the channel (Paynter et al., 2010), and we have previously shown that mutations that selectively destabilize the closed state (e.g., K80M) produce a decrease in pH sensitivity (Rapedius et al., 2007a). This suggests that a change in the relative energetic stability of these states can produce a shift in the pH-gating equilibrium and change the apparent pH sensitivity of the channel. We have therefore exploited this effect to explore the functional relationship between the different conformational states within the proposed Kir channel gating pathway.

In this study, we undertook an extensive scanning mutagenesis of Kir1.1 and measured the effect of these mutations on the pH-gating equilibrium. Our hypothesis was that by mapping many mutations with similar functional effects (e.g., destabilization of the open state) onto these different conformations of Kir1.1, we might gain a clearer insight into the state-dependent interactions that stabilize one specific structural state over another. We initially assumed that Kir1.1 channel gating can be described as a reversible transition between two structurally distinct states controlled by intracellular H^+^ (i.e., open and closed) and that the stability of each state will be influenced by interactions unique to each structural state. Consequently, when the channel moves between the open and closed conformations, the interactions and packing between these gating-sensitive residues will change. Thus, by mutating these residues to alanine (which will predominantly reduce interactions), we predicted that this might differentially affect the relative free energy of the closed versus open state. The aim of this approach was therefore to gain insight into the structural basis of the thermodynamic stability of the different crystallographic states within the proposed gating pathway.

To achieve this, we systematically mutated a major portion of the Kir1.1 channel (189 positions in total) and identified 49 mutations that markedly affected pH gating. These mutations were found to be in those regions predicted to undergo structural change during channel gating, thereby functionally validating this gating scheme. However, the most remarkable observation was that 95% of these mutations appeared to preferentially destabilize the open state due to their disruptive influence on a large state-dependent gating network and explain why so many mutations, including many disease-causing mutations, increase the pH sensitivity of Kir1.1. Importantly, these results also provide insight into the thermodynamic stability of these different gating states within the Kir channel gating pathway and the more general nature of the conformational transitions that occur in other allosteric proteins.

## Results

### Systematic Alanine Scanning Mutagenesis of Kir1.1

Assuming that a mutation does not directly affect the actual H^+^-sensing residue(s), then for a simple two-state gating scheme, any change in the H^+^ concentration required to half-maximally reduce channel activity (pH_0.5_) will be related to the change in the free energy difference between the open and closed states, i.e., ΔΔG_(open−closed)_ ≈2.3*RTn*(pH_0.5(WT)_ − pH_0.5 mutant_), where *n* is the Hill coefficient. However, this is most certainly an oversimplification as the pH gating mechanism is likely to involve multiple states. Furthermore, unlike voltage-gated potassium channels (Zagotta et al., 1994), there are currently no validated kinetic gating models for pH gating of Kir1.1, and so directly calculating ΔΔG_(open−closed)_ values from the shift in pH_0.5_ value is probably unjustified. Nevertheless, the direction of any change in pH_0.5_ value remains meaningful and will correlate with the direction of the free energy change. For example, a decrease in pH sensitivity will reflect an increase in ΔG_(open−closed)_, whereas an increase in pH sensitivity (i.e., a lower [H^+^] required to close the channel) will indicate a decrease in ΔG_(open−closed)._

**Figure 1:**
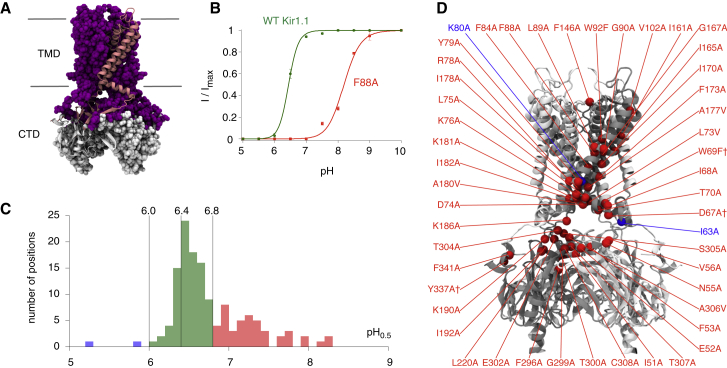
Mutations in Kir1.1 Preferentially Increase pH Sensitivity (A) The TMD and top half of the CTD were systematically mutated at a total of 187 positions (shown in magenta with a single monomer picked out in pink) and their pH_0.5_ values determined. (B) The F88A mutant substantially increases pH sensitivity compared to WT Kir1.1. Currents were recorded in giant excised patches over a range of pH values, allowing the pH_0.5_ value and Hill coefficient (*n*) to be measured. Data points represent mean ± SEM. (C) Mutations at 86 positions had relatively little effect on the pH_0.5_ value, leading to a shift of less than 0.4 pH units (green). Forty-seven mutants increased the pH_0.5_ by > 0.4 pH units (red), but only two mutants decreased the pH_0.5_ value by < 0.4 pH units (blue). No measurement of channel activity was possible for mutants at 52 positions (Table S1). (D) Mutations for which pH_0.5_ > pH 6.8 (red) and pH_0.5_ < pH 6.0 (blue) are mapped onto a single monomer (shown in gray) of a Kir1.1 closed-state model. †Indicates the coexpression with WT mRNA in a 1:1 ratio used to rescue the functional expression. See also Figure S1 and Movie S1.

Kir1.1 contains 391 amino acids; therefore, analyzing every single amino acid represents a major challenge. However, current models of Kir channel gating indicate that the largest structural changes occur primarily within the TMD and at the interface between the TMD and the CTD. Consequently, we restricted mutagenesis to these regions, and the residues chosen are highlighted in Figure 1A (for details, see Table S1 available online). This selection represents 187 residues that were then individually mutated to alanine [or to valine if the wild-type (WT) sequence was already alanine] and their impact on intracellular pH gating determined. An example of one mutant (F88A) that shifts the pH_0.5_ from pH 6.4 ± 0.1 (for WT Kir1.1) to pH 8.3 ± 0.1 is shown in Figure 1B. This mutation involves a residue within transmembrane helix 1(TM1) that is clearly nontitratable, and the approximate 80-fold change in [H^+^] required to close the channel must therefore reflect changes in the relative stability of the open versus closed state.

In cases where the alanine mutants within the TM helices were nonfunctional, a more conservative amino acid substitution was also examined; when this still failed to produce functional channels, a 1:1 coexpression with wild-type Kir1.1 mRNA was finally used in an attempt to rescue channel activity. This approach maximized the data available from this systematic screen and led to pH_0.5_ values being obtained from 135 positions (Table S1). Perhaps not surprisingly, of the 52 nonfunctional mutants, many were found to be clustered in or near the selectivity filter and were not examined further.

### Biased Effect of Mutations on pH Sensitivity of Kir1.1

To summarize this large data set, the 135 functional mutants were categorized according to their relative effect on the pH_0.5_ value (Figure 1C). Mutations at 86 positions were found to have relatively little or no effect on the pH_0.5_ value (i.e., they fell within pH_0.5_ = 6.0–6.8 compared to wild-type Kir1.1, pH_0.5_ = 6.4) (Figure 1C), and many of these residues were located on the outer surface of the channel, in particular the outer face of TM1 (Movie S1). However, of most interest was the startling observation that out of the 49 mutations that shifted the pH_0.5_ by > 0.4 pH units, 47 (i.e., > 95%) were shifted into the alkaline range (pH_0.5_ > pH 6.8) (Figure 1C), whereas only 2 mutations produced a reduction in pH sensitivity (pH_0.5_ < pH 6.0), both of which have been identified previously (K80A and I63A). This extreme functional bias is clearly visible in the histogram in Figure 1C.

The pH inhibition of wild-type Kir1.1 has a Hill coefficient of 2.5 ± 0.2, but although the majority of the 49 mutations that changed the pH_0.5_ had relatively little effect on the Hill coefficient (Δn < 0.5), 14 mutations were found to increase the Hill coefficient (Δn > 0.5), while 10 reduced the Hill coefficient by more than 0.5 units. In this latter category, four mutations (at positions 67, 69, 70, and 220) had a particularly pronounced effect (Δn > −1), and a structural interpretation of this finding is discussed later. However, because these changes in the pH_0.5_ and Hill coefficient do not appear to be correlated (Figure S1) and because mechanistic interpretation of the Hill coefficient remains controversial (Weiss, 1997), only the observed changes in pH_0.5_ were considered for further analysis.

### Preferential Destabilization of the Open State

As stated above, 47 of the 49 mutations that altered the pH sensitivity caused an increase in pH sensitivity. Therefore, how can we explain such a large bias in the effect of these mutations? The majority of the pH_0.5_ > 6.8 residues are nontitratable and must therefore have an indirect effect on pH gating. In any protein structure the most likely effect of a mutation is to destabilize that particular structure (Yifrach et al., 2009). Consequently, the biased effect of these mutations suggests that their disruptive effect is far greater on the open state than on the closed state. This will make it easier for H^+^ to shift the equilibrium toward the closed state, thus causing an alkaline shift in the pH sensitivity. If this is the case, then these pH_0.5_ > 6.8 residues may be involved in state-dependent interactions that preferentially stabilize the open state, and their perturbatory effect may therefore be related to their physical proximity in the open state.

To determine whether any state-dependent physical interactions exist between these pH_0.5_ > 6.8 residues, we generated homology models of Kir1.1 in the proposed closed, pre-open, and open states. As a template for the closed state, we used the Kir2.2 structure (Protein Data Bank [PDB] 3JYC) (Tao et al., 2009), while the recent PIP_2_-bound structure (PDB 3SPI) (Hansen et al., 2011) was used as template for the pre-open state. To model the open state (i.e., where the bundle crossing gate is wide enough to allow K^+^ permeation), we used a symmetrized model of Kir3.2 in a potentially open conformation (open-Kir3.2 model) where all four subunits have been modeled in the open state (Whorton and MacKinnon, 2011). Full details of how these models were constructed, their relative geometry, the pore radius, and the height and twist of the CTD are described in Table S2.

### Assembly of a State-Dependent Gating-Sensitive Network

**Figure 2:**
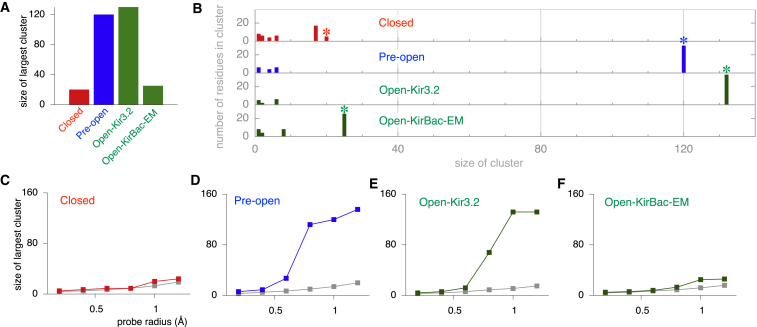
Gating-Sensitive Residues Interact in a State-Dependent Network (A) The size of the largest cluster formed by the 47 pH_0.5_ > pH 6.8 residues determined in the closed, pre-open, open-Kir3.2, and open-KirBac-EM models of Kir1.1. Residues interactions were defined as described in the Supplemental Experimental Procedures by using a probe radius of 1.0 Å. (B) Distributions in cluster size obtained for the indicated Kir1.1 models. The largest cluster in each model is indicated by an asterisk. Randomly selected residues exhibit clusters of ≤ 10 in all models (see also Figure S3). (C–F) The size of the largest cluster of pH_0.5_ > pH 6.8 interacting residues plotted against the probe radius used to define a packing interaction in the indicated Kir1.1 models (colored squares); notice that no large clusters are formed in the closed and open-KirBac-EM models residues, but large clusters appear as the probe radius increases to 0.8–1.0 Å in the pre-open and open-state models (see also Figure S4). Also shown in gray squares is the average size of the largest cluster for an ensemble of 100 models where the 47 positions were chosen randomly out of the 187 investigated positions; notice no larger clusters appear in any of the Kir1.1 models (see also Figure S5).

The 47 pH_0.5_ > 6.8 residues were mapped onto the three different structural models of Kir1.1, and any physical interactions between the residues were then scored. Because the channel is tetrameric, a total of 188 residues (i.e., 4 × 47) were examined for potential intersubunit or intrasubunit interactions, and if ≥2 residues were found to interact, then this was defined as a “cluster.” In addition to counting H bonds and salt bridges, we determined how close these residues pack by using a probe of 1.0 Å radius rolled over the side chain of each residue to determine its surface area. This also reflects its accessible surface area and contribution to the hydrophobic effect (Richards, 1977). This process was then repeated for each pair of residues (including all possible intrasubunit and intersubunit combinations), and if the total surface area of the pair was less than the combined surface area of each separate residue, then an interaction was scored (see Supplemental Information for further details of the methods involved and Figure S2). We found that the size and distribution of these clusters varied dramatically between the different models of Kir1.1. In the closed state, only a series of smaller clusters was observed, with the largest involving only 20 residues (Figures 2A and 2B). By contrast, in the pre-open and open-state models, almost two-thirds of the pH_0.5_ > 6.8 residues were found to assemble into a single large cluster or “network” involving between 120 and 132 residues (Figures 2A and 2B).

Clearly, the outcome of such an analysis will be dependent upon the radius of the probe used: if it is too small, then an analysis of a rigid model will not take into account the thermal motion of the side chains. Likewise, if the probe is too large, then many false positives could arise. To address this, we examined how the size of the largest cluster varied with the radius of the probe used. In the closed state no increase in network size was seen when the probe radius was varied between 0.2 and 1.2 Å (Figure 2C). However, for both the pre-open and open-state models, there was a sudden and dramatic increase when the probe radius was increased from 0.6 to 0.8 Å (Figures 2D and 2E). Intriguingly, the crystallographic B factors of several high-resolution ion channel structures indicate that the thermal motion of residues within these structures leads to atomic fluctuations in the range of 0.8 to 1.0 Å (Halle, 2002; Noskov et al., 2004). Therefore, using a probe radius of 1.0 Å takes such thermal fluctuations into account, which suggests this is a reasonable approach for determining the physical connectivity between residues (see Figure S4 and Supplemental Information for further details).

### Specificity of Network Assembly

When compared to the closed state, the pre-open and open conformations are more compact due to the upward movement of the CTD and interaction with the TMD. So how can we be sure that the apparent assembly of so many pH_0.5_ > 6.8 residues into a single large network within the pre-open and open states is mechanistically meaningful and not simply a consequence of the more compact nature of these structures?

To rigorously examine this, we made 100 copies of each model, where 47 positions were randomly chosen from those 187 residues mutated within each tetramer, and the same analysis was then repeated. These results, plotted as a function of probe radius (Figures 2C–2E; Figure S5) show that no large clusters of interactions appear in any of the structural states, even when the probe radius was increased up to 1.2 Å. As a further control, we also generated another open-state model (open-KirBac-EM model) based upon very low-resolution 2D electron microscopy projection images of a prokaryotic Kir channel (Domene et al., 2005), and the same analysis was then applied. Despite its overall similarity in shape and pore diameter, this low-resolution model is markedly different from more recent Kir/KirBac channel open-state crystal structures and generated cluster sizes no different from randomly selected residues (Figures 2A and 2F). Together, these controls suggest that the formation of a single large cluster of interacting residues in the pre-open and open state is specific to the selection of these 47 pH_0.5_ > 6.8 residues and that with the exception of the open-KirBac-EM model, the structural models used in this analysis represent functionally relevant conformations within the Kir1.1 channel gating pathway.

### Structural Basis of the pH-Dependent Gating Step

**Figure 3:**
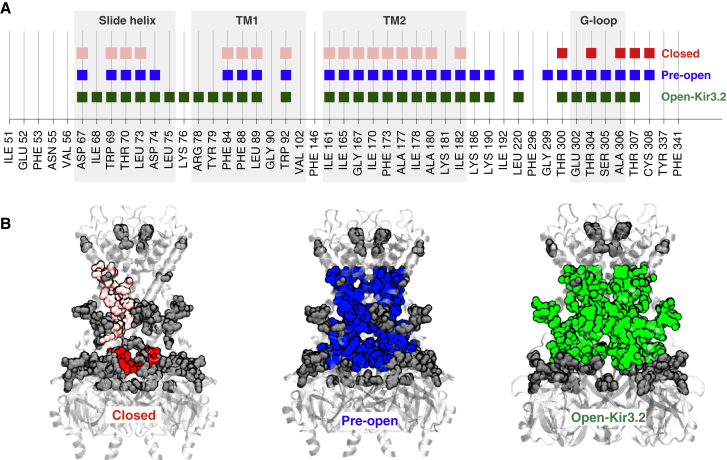
Assembly of a Large Gating Network in the Pre-Open and Open States (A) All 47 identified pH_0.5_ > 6.8 residues are shown on the bottom axis, and those that belong to a network of intermediate or large size are identified by colored squares. (B) The same residues that form the networks are mapped onto the appropriate structural model of Kir1.1 by using the same coloring scheme. Other pH_0.5_ > 6.8 residues are shown in dark gray. In the closed model there are five networks of intermediate size: one involving the G loop of each monomer (shown in red) and four identical clusters that connect the TM1, TM2, and slide helices (only one is shown for clarity, in pink). As the CTD moves upward, these smaller clusters fuse together in the pre-open model, forming a single large network spanning all four monomers (blue). This connects the TMD to the G loop and the CTD, and almost all these residues remain connected in the open state (green). See also Movie S2 and Figure S6.

Comparison of these different gating conformations also revealed a high degree of overlap in the identity of gating-sensitive pH_0.5_ > 6.8 residues found in each network and provides some insight into how this network might assemble (or break down) as the channel moves back and forth between these different conformations (Figure 3A). In both the pre-open and open states, this consensus involves residues from the slide helix, TM1 and transmembrane helix 2 (TM2) of the TMD, and also the G loop, which all then become fused together into a single large intersubunit and intrasubunit network. The residues involved do not differ substantially between these two states. Interestingly, many of these same residues also contribute to clusters in the closed state. However, in the closed state the single large network is broken down into a series of smaller intrasubunit and intersubunit clusters (Figure 3B; Movie S2). In the closed state the largest individual cluster is located within the CTD where 5 residues within each of the 4 G loops contribute to a cluster of 20 residues. The second largest network in the closed state involves 4 identical (but physically separate) clusters of 17 residues within the TMD. Comparison of these different open and closed conformations indicates that the upward movement of the CTD allows assembly of these five preexisting clusters into the single large network in the pre-open and open states (Figure 3B; Movie S2).

Importantly, the fact that this large gating-sensitive network does not appear to differ substantially between the pre-open and open states indicates that the greatest change in physical connectivity between the pH_0.5_ > 6.8 residues occurs during the transition between the closed and pre-open states. Therefore, it seems likely that this transition between closed and pre-open states represents the pH-dependent gating step most affected by the pH_0.5_ > 6.8 residues (see Discussion).

### Double Mutant Cycle Analysis Reveals Long-Range Allosteric Coupling

Double mutant cycle analysis can be used to determine the independence or dependence of the functional effects of two or more individual mutations on the function of a protein (Maksay, 2011; Yifrach et al., 2009). For example, if two separate regions both undergo independent, localized conformational changes that contribute to pore opening, then mutations in these two regions should have independent (i.e., energetically additive) effects. Alternatively, a larger, more concerted conformational change might involve coupling between distant regions of the protein. In this case, even though far apart, mutations in these two regions would have nonadditive effects on gating (Yifrach and MacKinnon, 2002). Based upon these assumptions, our prediction was that combinations of mutations within this gating network would show energetic coupling even though they might be far apart within the structure.

**Figure 4:**
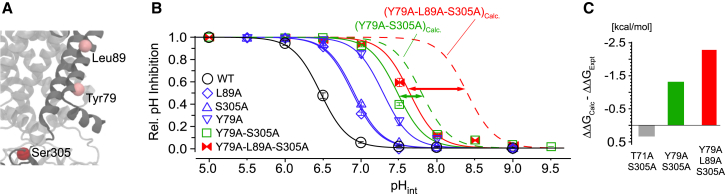
Mutant Cycle Analysis Reveals Long-Range Thermodynamic Coupling (A) Location of network mutations L89A, Y79A (TM1), and S305A (G loop) in the open-Kir3.2 model. (B) The pH sensitivity of WT and indicated mutants. Calculated dose-response curves for double and triple mutants are also shown assuming no thermodynamic coupling (see Experimental Procedures). Data points represent mean ± SEM. (C) Thermodynamic coupling between indicated mutations was determined as the difference between the calculated ΔΔG_Calc_ and experimental ΔΔG_Expt_ values for indicated double and triple mutants. Note that thermodynamic coupling increases with the addition of L89A on Y79A-S305A. The T71A mutant is used as a control because this mutation is not within the network and exhibits WT pH sensitivity. The T71A-S305A double mutant exhibits no coupling.

To test this we combined two different mutations in TM1 (Y79A: pH_0.5_ = 7.3 ± 0.1, n = 2.5 ± 0.1; L89A: pH_0.5_ = 6.9 ± 0.1, n = 2.6 ± 0.1) with a mutation within the G loop (S305A: pH_0.5_ = 6.9 ± 0.1, n = 2.5 ± 0.1) (Figure 4A). If their effect on pH gating is additive (i.e., not energetic coupled), then the respective double mutants should have pH_0.5_ values corresponding to the sum of the pH shifts of the individual mutants. For example, assuming there is no change in the Hill coefficient, then for the Y79A-S305A double mutant, a pH_0.5_ value of 7.8 would be expected if the mutations were purely energy additive. However, we found that the Y79A-S305A Kir1.1 channels had a pH_0.5_ value of 7.5. Moreover, if we added a third mutation (L89A) to the Y79A-S305A double mutant, the pH_0.5_ was shifted to 7.6, whereas a value of 8.3 would be expected if these three mutations were not coupled (Figure 4B). In more quantitative terms the magnitude of nonadditivity of the double mutants can also be calculated (see Experimental Procedures). The resulting ΔΔG_(open−closed)_ values for Y79A-S305A and Y79A-L89A-S305A were −1.3 kcal/mol and −2.3 kcal/mol, respectively. However, when S305A within the G loop was combined with a control mutation that does not alter pH gating (T71A), then the ΔΔG_(open−closed)_ value was marginal (0.3 kcal/mol). Comparison between the experimentally determined (ΔΔG_Expt_) and calculated (ΔΔG_Calc_) values demonstrates that residues within the gating network can couple energetically over large distances (Figure 4C and Discussion).

### Clustering of Mutations that Reduce the Hill Coefficient

**Figure 5:**
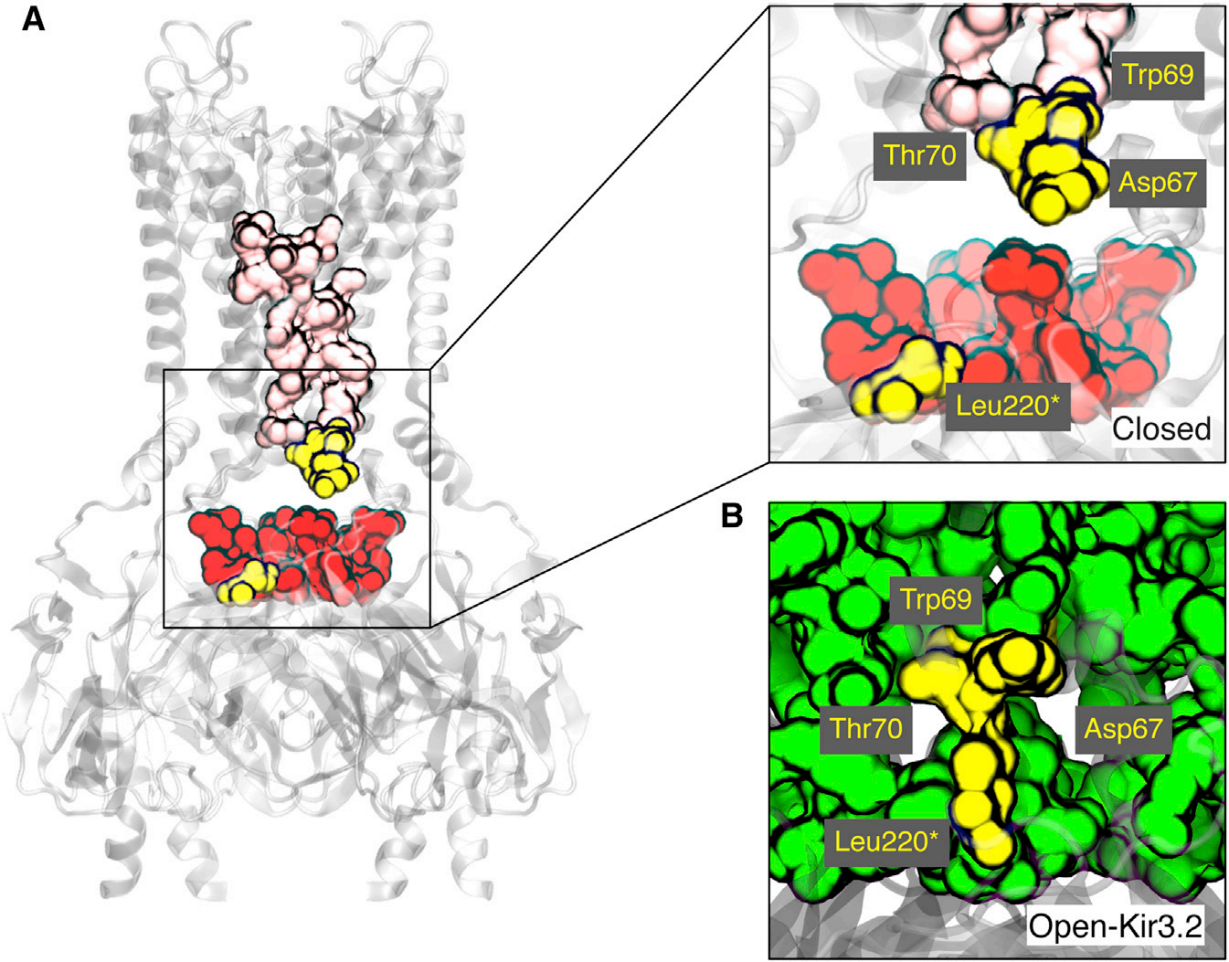
Intersubunit Interactions Affect Subunit Cooperativity Asp67, Trp69, and Thr70 on the slide helix and Leu220 on the CD loop of the CTD all shift the pH_0.5_ > 6.8 and also significantly reduce the Hill coefficient (Δn > −1). They are separated by a large distance in the closed model (A), but come together in the open-Kir3.2 model (B) to form a connection between the TMD and the CTD. For context, the large networks are shown as in Figure 4B. Leu220 is indicated by an asterisk to denote this residue belongs to an adjacent subunit and represents part of an intersubunit interaction (see also Figure S1).

There is little functional correlation between mutations that affect pH_0.5_ and their effect on the Hill coefficient (Figure S1). Furthermore, those mutations that increased the Hill coefficient appear to be scattered throughout the Kir1.1 structure. However, examination of the four mutations (D67A, W69F, T70A, and L220A) that produced the largest reduction in Hill coefficient (Δn > −1) appears to provide some mechanistic insight. Asp67, Trp69, and Thr70 are located within the slide helix at the TMD-CTD interface and are involved in the fusion of several smaller clusters within the closed state into the single large network seen in the pre-open and open states (Figure 5). Furthermore, Leu220 is located on the CD loop of the CTD and comes into close proximity to Thr70 when the channel switches from the pre-open state to the open state. Thus, the marked reduction in the Hill coefficient observed for these four mutations appears to correlate with their involvement in a state-dependent intersubunit interaction and also suggests that this region may be critical for subunit cooperativity during the pore opening step.

## Discussion

In this study we have revealed a large number of “gating-sensitive” residues that have a marked preference for destabilization of the open state. Furthermore, reconstruction of a structural gating pathway for Kir1.1 also revealed that most of these gating-sensitive residues assemble into a large physically connected network found only in the open and pre-open states. Mutagenic perturbation of this state-dependent network therefore provides a straightforward explanation for the increase in pH sensitivity observed in these mutants and suggests that intracellular pH gating may control the transition between the pre-open and closed conformations.

### Structural Optimization of the Open State

One of the most remarkable findings of this study was the functionally biased effect of mutations on the pH gating of Kir1.1, i.e., 47 of the 135 functional mutations increased the pH sensitivity, whereas only 2 mutations decreased pH sensitivity. Assuming that the calculated pH_0.5_ value broadly reflects the difference in free energy between the open and closed states (and that mutations generally have a destabilizing effect on protein structures), we therefore conclude that the open state is more sensitive to mutagenic perturbation than the closed state. Interestingly, scanning mutagenesis of the TM helices of the voltage-gated *Shaker* channel revealed a different picture (Yifrach and MacKinnon, 2002). In that study, most mutations preferentially destabilized the closed state, suggesting that it is intrinsically more stable than the open state. This outcome seems intuitively reasonable as we would expect evolution to have optimized the principal physiological state of the channel, i.e., open for Kir1.1 (at physiological pH), but closed for a voltage-gated K^+^ channel (at the resting membrane potential).

### Assembly of a State-Dependent Gating Network

Our analysis of the physical interactions between the 47 gating-sensitive residues found that most are involved in either intersubunit or intrasubunit interactions with other pH_0.5_ > 6.8 residues. However, although many of these interactions occurred in all three different structural states (i.e., closed, pre-open, and open), we found a remarkable difference in the relative size of the networks involved; in the closed state, only a series of smaller clusters are observed, whereas in the pre-open and open-state models a large cluster of up to 132 residues is found. The principal structural reason for this dramatic increase in network size is the upward movement of the CTD as it engages with the TMD in the pre-open state. This transition then fuses the smaller preexisting clusters found in the closed state into a larger single network of intersubunit and intrasubunit interactions that spans across the membrane from the G loop up toward the selectivity filter (Figure 3; Movie S2).

### Network Connectivity Determines State Stability and Mutagenic Sensitivity

**Figure 6:**
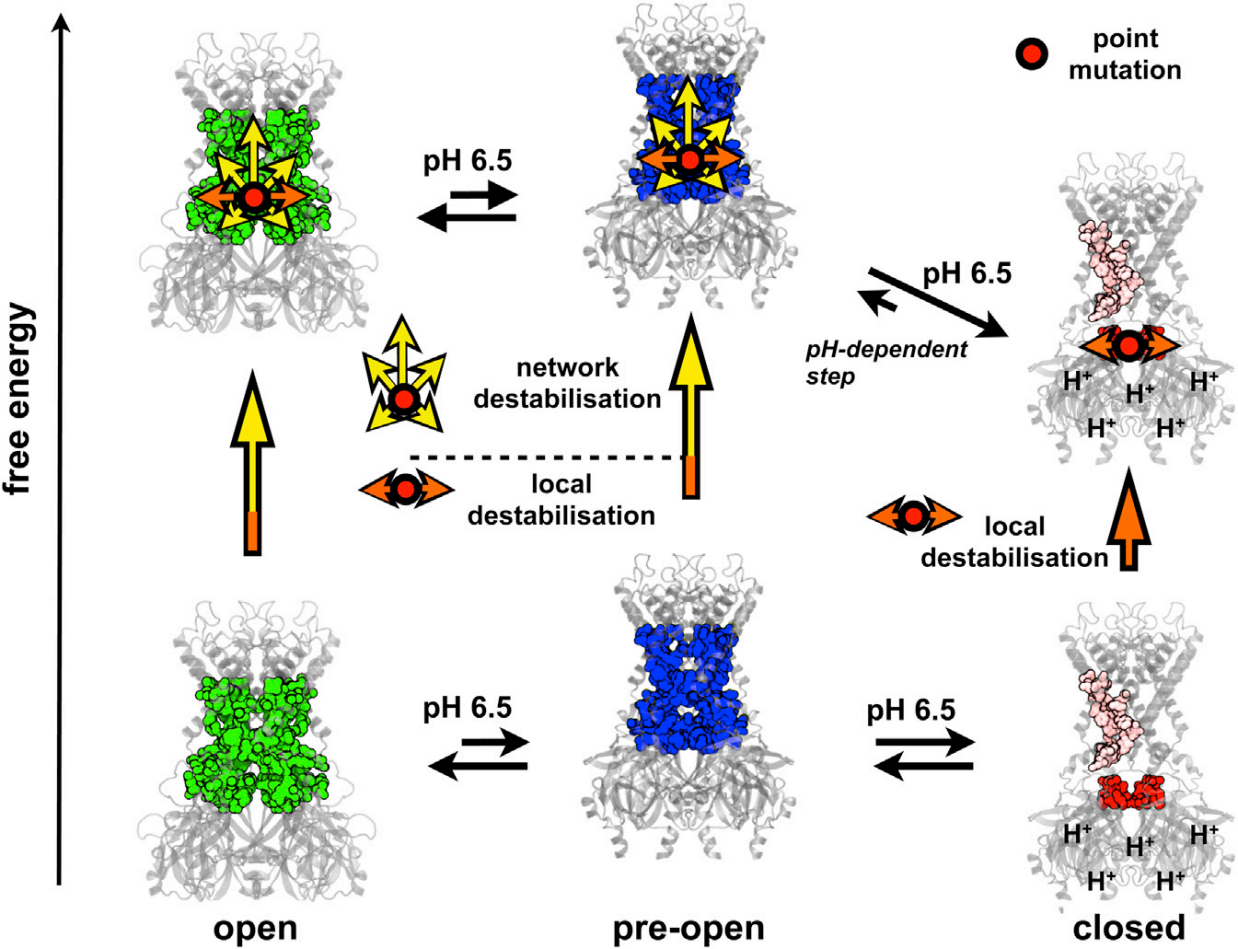
Impact of a Network Mutation on the Gating of Kir1.1 Cartoon depicting the assumed free energies of the different conformational states of Kir1.1 at pH 6.5, i.e., the equilibrium point where the open and close states are about equally populated (bottom row). The gating network residues are highlighted in green (open state), in blue (pre-open state), and in red (the closed state). Note that the gating network in the closed state is fragmented into smaller clusters. Mutation of a gating network residue (red dot) will have a local destabilizing effect (local destabilization, orange arrows) on all states. However, it will have a larger effect on the open and pre-open states due to greater connectivity of the gating network (network destabilization, yellow arrows) in these states compared to the closed state. This raises the free energy of the pre-open and open states relative to the closed state and leads to a redistribution in the relative population of states. The closed state now becomes more energetically favorable and therefore more frequently populated, thereby explaining the increased pH sensitivity observed for mutations within this network.

How does the apparently biased effect of mutations on the pH sensitivity of Kir1.1 correlate with their assembly into a large gating-sensitive network in the pre-open and open states? It is possible that the local impact of mutating these gating-sensitive residues might not exhibit any state dependence, thus the local destabilization might be similar in all states. However, it is well established that residues can be energetically coupled over large distances in a protein (Sadovsky and Yifrach, 2007; Yifrach et al., 2009). Thus, in addition to any local effect, a mutation at one position can affect many other distant residues within the protein. We therefore propose that the enhanced connectivity of this network in the open (and pre-open) state enhances the perturbatory effect of a single mutation by allowing it to spread much wider than in the smaller more fragmented networks of the closed state, thereby causing an increased level of network destabilization as illustrated in Figure 6.

This concept is supported by our finding that distant residues within the gating network show strong thermodynamic coupling (Figure 4). Although such coupling could potentially result in either an increase or decrease in the combined effect, the negative values observed for this thermodynamic coupling are consistent with the concept of the gating network stabilizing the open (and pre-open) state, but not the closed state because an existing mutation within the network will reduce the destabilizing effect of a second (or even third) additional mutation. In other words, if the network is already destabilized by mutagenesis, then the impact of further mutations on the open state will be reduced and become more similar to the impact of the mutation on the closed state. As a consequence, the impact of the mutation on the state equilibrium (i.e., pH sensitivity) vanishes.

It therefore seems reasonable to propose that the thermodynamic coupling we observe is physically transmitted via interactions within the network. This not only provides a structural explanation for the observed mutagenic sensitivity of the Kir1.1 open state but may also highlight a general property of residues within allosteric proteins, i.e., their state-dependent physical connectivity.

### Functional Validation of a Gating Pathway

X-ray crystallography has resolved the structure of Kir2.2 in three different structural states, i.e., the closed, pre-open, and open states. However, it is not clear per se whether these crystallographically defined structures are physiological relevant within the native membrane. For instance, how do we know whether the closed state is similar to the native closed state of a Kir channel other than its narrow pore diameter?

The data we present here provide functional validation of the closed-state and pre-open-state conformations as well as the proposed open state. This conclusion is supported by the following observations. First, all of the gating-sensitive residues are located within those regions that undergo structural changes between the different crystallographic states. Second, our method of determining this gating network was sensitive to both the nature of the structural template used (no such network was found in the low-resolution open-KirBac-EM structure) as well as the exact selection of residues (100 different sets of randomly selected residues failed to reproduce this network). In other words, our functional data validate these structural models and give confidence that they represent a plausible Kir channel gating pathway.

### Pathophysiological Implications

Our results may also explain why so many mutations in Kir1.1 give rise to type II Bartters syndrome. This salt-wasting renal tubulopathy is caused by a variety of mutations in Kir1.1 that result in a loss of function (Hibino et al., 2010). Intriguingly, a number of Bartters mutations have been shown to produce an alkaline shift in pH sensitivity resulting in a loss of function at physiological pH, and several of these mutant residues (e.g., Asp74, Ala177, and Ala306; Hibino et al., 2010) are found within the network of gating-sensitive residues identified in this study. Their effects on pH sensitivity are therefore likely to be the result of destabilizing this gating network. Furthermore, loss of function mutations due to an alkaline shift in pH_0.5_ in the related Kir4.1 channel have also been shown to underlie another tubulopathy (SeSAME/EAST syndrome) (Bandulik et al., 2011), and it is possible that a similar network effect may also exist in this channel.

In summary, our systematic scanning mutagenesis approach now provides an insight into the structural and energetic landscape of the Kir1.1 channel gating pathway. This integrated approach of computational and functional analysis has identified a large network of physically connected residues that preferentially stabilizes the pre-open and open states of the channel and highlights the structural basis of the pH-dependent gating transition. Importantly, this analysis would not have been possible without the comprehensive nature of the scanning mutagenesis undertaken. Had our analysis been restricted to fewer residues or one particular conformation, then structural interpretation of these mutations would have been limited. Our results also provide evidence that the physical network connectivity of state-sensitive residues may represent a structural mechanism for thermodynamic coupling between distant sites in a protein. Furthermore, they suggest that the thermodynamic consequences of mutagenic perturbation in a particular state are related to the degree of network connectivity. Further studies will have to show whether this is a general property of allosteric proteins, but we anticipate that substantial (i.e., global) structural changes will be required to cause large changes in physical network connectivity as seen here in Kir channel gating. It is inevitable that more ion channel structures in multiple different conformational states will become available. This study therefore demonstrates that similar comprehensive analytical approaches may emerge as a worthwhile approach to better understand the thermodynamic consequences of structural changes in other allosteric proteins.

## Experimental Procedures

### Molecular Biology and Electrophysiology

Mutagenesis of rat Kir1.1a in the pBF oocyte expression vector was performed using the QuikChangeII system (Agilent). The mRNAs were synthesized using the SP6 mMESSAGE mMACHINE kit (Ambion). Manually defolliculated *Xenopus* oocytes were injected with 2–5 ng mRNA, and the intracellular pH sensitivity was determined from giant patches in inside-out configuration under voltage clamp conditions 3–7 days after mRNA injection. Pipettes were made from thick-walled borosilicate glass, had resistances of 0.3–0.9 MΩ (tip diameter of 5–15 μm), and were filled with (in mM, pH adjusted to 7.2 with KOH) 120 KCl, 10 HEPES, and 1.8 CaCl_2_. Currents were sampled at 1 kHz with an analog filter set to 3 kHz (−3dB). Solutions were applied to excised patches via a multibarrel pipette and had the following composition (in mM): 120 KCl, 10 HEPES, and 2 K_2_EGTA, adjusted to the appropriate pH with HCl. The pH dose-response curves were determined as described above (Rapedius et al., 2007b). For the thermodynamic coupling analysis shown in Figure 5, ΔΔG_Calc_ values were calculated assuming that single mutations are energetically additive when combined, i.e., ΔΔG_(double mutation)Calc_ = ΔG_(first mutation)_ + ΔG_(second mutation)_ and ΔΔG_(triple mutation)Calc_ = ΔG_(first mutation)_ + ΔG_(second mutation)_ + ΔG_(third mutation)_. The ΔG_Expt_ values were calculated from the experimentally determined values according to the equation ΔΔG_Expt_ = 2.3 *RTn* [pH_0.5(mutant-double/triple)_ − pH_0.5(WT)_]. The Hill coefficient (*n*) was 2.4 for WT and between 2.3 and 2.6 for all mutants; therefore, it was set to 2.4 for all fits.

### Homology Modeling and Analysis

The closed and pre-open models of Kir1.1 were built using Modeler 9v8 (Sali and Blundell, 1993) primarily from structures of cKir2.2 (Hansen et al., 2011; Tao et al., 2009). The open-Kir3.2 and open-KirBac-EM models were built using open structures of Kir3.2 and a model of KirBac1.1 built using low-resolution EM maps (Domene et al., 2005; Hansen et al., 2011). The coordinates of the models along with more details of the process, including a sequence alignment, can be found in the Supplemental Information. The measured values of the pH_0.5_ and Hill coefficient are stored in the BETA and OCCUPANCY fields, respectively. The 47 pH_0.5_ > 6.8 residues were then mapped onto these structures, and hydrogen bonds and salt bridges were detected by using VMD (Humphrey et al., 1996). In addition, residues were assessed as packing against one another by using a probe of variable radius. More details on this along with a discussion of relating the radius of the probe to Debye-Waller B factors can be found in the Supplemental Information. Graphs were then constructed using NetworkX (Hagberg et al., 2008), where residues were nodes and interactions between them formed edges. The distribution of cluster sizes was then analyzed and plotted.

## Supplemental Information

Document S1. Supplemental Experimental Procedures, Figures S1–S6, and Table S2

Table S1. Large Data Set, Related to Figure 1 This is contained within a separate file and lists the mutations analyzed and their effect on the measured pH_05_ and Hill coefficients. If a simple alanine mutation led to a channel with no expression, a more conservative mutation was tried. If this also failed, in some cases a coexpression with wild-type was then attempted. Standard errors were calculated in the usual way and are also included.

Movie S1. The Model of Closed Kir1.1, Related to Figure 1 The closed model of Kir1.1 is rotated, showing (A) which positions were mutated (grey), (B) where mutation resulted in no functional activity (orange), (C) where mutation changed the pH_05_ by less than 0.4 pH units (green), (D) where pH_05_ < 6.0 (blue), and (E) where pH_05_ > 6.8 (red, 47 positions).

Movie S2. Five Networks Identified in the Closed State Fuse Together, Forming a Single Network in the Pre-Open state, which Persists in the open State, Related to Figure 3 The five largest networks (four identical in the TMD and one connecting all the G loops in the CTD) in the closed state are shown on the model using space-filling spheres. As an aid to understanding, a morph from the closed state to the pre-open state is shown, demonstrating how the conformational changes cause these networks to fuse together and form a single large network. This network remains approximately the same, even when the channel undergoes further conformational changes and opens. This is illustrated by a morph from the pre-open to open state (open-Kir3.2 model).

Document S2. Article plus Supplemental Information
