# Supplementary material for: State-Dependent Network Connectivity Determines Gating in a K+ Channel
Source: Structure. 2014 Jul 8;22(7):1037–46. doi: 10.1016/j.str.2014.04.018 (PMC4087272; doi:10.1016/j.str.2014.04.018)
Supplement: Document S1. Supplemental Experimental Procedures, Figures S1–S6, and Table S2 [file mmc1.pdf]

Structure, Volume *22*

Supplemental Information

State-Dependent Network Connectivity Determines Gating in a K^+ Channel

Murali K. Bollepalli, Philip W. Fowler, Markus Rapedius, Lijun Shang, Mark S.P. Sansom, Stephen J. Tucker, and Thomas Baukrowitz

State-Dependent Network Connectivity Determines Gating in a K^+ Channel – Supplementary Material

Murali K. Bollepalli*[1,6], Philip W. Fowler*[2], Markus Rapedius[1], Lijun Shang[3,5], Mark S. P. Sansom[2,4], Stephen J. Tucker[3,4] and Thomas Baukrowitz[1]

1. Physiological Institute, Christian-Albrechts University, 24118 Kiel, Germany
2. Department of Biochemistry, University of Oxford, Oxford, OX1 3QU, UK
3. Clarendon Laboratory, Department of Physics, University of Oxford, Oxford, OX1 3PU, UK
4. OXION Ion Channel Initiative, University of Oxford, Oxford, OX1 3PT, UK
5. Present address: School of Medical Sciences, Bradford University, Bradford, BD7 1DP, UK
6. Present address: Department of Physiology, Development and Neuroscience, University of Cambridge, Cambridge, CB2 3EG, UK

MKB and PWF contributed equally to this study.

Correspondence should be addressed to TB[1] or SJT[2].

Contents

1 Supplementary Methods **3**

1.1 Construction of closed, pre-open and open Kir1.1 homology models 3

1.2 Characterization of the closed, pre-open and open Kir1.1 models 3

1.3 Analysis of the closed, pre-open and open Kir1.1 models 4

1.4 Relating the probe radius to the predicted atomic root mean square fluctuation 5

2 Supplementary Tables **6**

3 Supplementary Movies **6**

4 Supplementary Figures 1–6 **6**

[1]t.baukrowitz@physiologie.uni-kiel.de

[2]stephen.tucker@physics.ox.ac.uk

List of Figures

S1 Lack of clear correlation between the $pH_{0.5}$ and the Hill coefficients (refers to Figure 1 and Figure 5). 10

S2 Schematic illustrating how homology models were built and analysed (refers to Experimental Procedures and Figure 2) 11

S3 Large clusters form in the Pre-open and Open-Kir3.2 models (refers to Figure 2B) 12

S4 Average RMSFs predicted from Debye-Waller crystallographic *B*-factors for three high resolution ion channel structures (refers to Experimental Procedures and Figure 2B) 13

S5 Clustering of $pH_{0.5} > 6.8$ positions (refers to Experimental Procedures and Figures 2C-F) 14

S6 Alignment used to build the homology models (refers to Figure 3) 15

1 Supplementary Methods

1.1 Construction of closed, pre-open and open Kir1.1 homology models

We built a closed, a pre-open and two open homology models of Kir1.1 using the available experimental structures of inwardly-rectifying potassium channels. The closed and pre-open models of Kir1.1 used the structures of chicken Kir2.2 without[1] (PDB:3JYC, resolution 3.1 Å) and with[2] PIP_2 (PDB:3SPI, resolution 3.3 Å) bound. One of the two open homology models used the structure of Kir3.2[4], whilst the other was based on and a published model of KirBac1.1[5].

The crystallographic unit cell of R201A Kir3.2 with PIP2 bound (PDB:3SYQ, resolution 3.5 Å) contains two monomers, one with PIP_2 bound and one without, resulting in a tetramer with only two-fold symmetry[4]. We used a symmetric tetramer with four-fold symmetry built using only the more open PIP_2-bound monomer coordinates supplied by the MacKinnon laboratory. Neither this structure nor the model of KirBac1.1 derived from 2D electron microscopy data[5] are complete templates for Kir1.1 and hence the Open-Kir3.2 and Open-KirBac-EM models required multiple templates (**Table S2**). The N-terminus of the Open-Kir3.2 model (residues 49-67 incl., Kir1.1 numbering) was modelled using the more open chimeric Kir/KirBac structure (PDB:2QKS, resolution 2.2 Å)[6]. For both models the long extracellular loop present in Kir1.1 (residues 104-120 incl.) was modelled using the closed structure of chicken Kir2.2 (PDB: 3JYC)[1]. The multiple alignment between Kir1.1 and all the homologs is shown in Figure S6. The quality of the alignment is good with many conserved sequences in both the pore and C-terminal domains.

We then used Modeller 9v8[7] to build the four homology models of Kir1.1. If more than one homolog was used, these were first structurally aligned using VMD[8] before being input into Modeller. The fragments were structurally aligned using the last or first 4 residues. All numbering below is for Kir1.1. Modeller enforced both the disulphide bond between Cys-121 and Cys-153 and the salt bridge between Glu-137 and Arg-147. The slide (residues 64-74), M1 (residues 77-104) and M2 (residues 154-183) helices were all constrained to be helical and finally the four chains were also constrained to be symmetrical. Fifty models were built and the best one was selected by examining both the reported energy and the number of residues in disfavoured and disallowed regions of the Ramachandran plot, as assessed by the RAMPAGE server[9]. Hydrogens were then added using the pdb2gmx module of GROMACS[10]. Each model of Kir1.1 contained at a minimum residues 49-350.

1.2 Characterization of the closed, pre-open and open Kir1.1 models

But which model is the most open at the helix bundle crossing and how do the positions of the C-terminal domain change? To provide an approximate answer to these questions we measured a series of parameters for each model

given in Table S2. These are (i) the distance between the A180 Cα atoms in TM2, (ii) the average pore radius and the (iii) twist and (iv) height of the CTD. We chose A180 as it is the equivalent residue to T112 in KcsA and so enables comparison with the range of open KcsA structures[11]. The distance between the A180 Cα atoms therefore gives a crude estimate of how far apart the M2 helices are and therefore how open is the helix bundle crossing. The average pore radius provides a more accurate assessment; first how the pore radius varies along the channel is estimated by HOLE[12]. We define the average pore radius as the average of the pore radius over a 7 Å window centered on the position of Cα of A180. This value can be compared to theoretical studies of hydrophobic pores[13]. The values suggest that both the helix bundle crossing of the closed and pre-open models of Kir1.1 are in all likelihood impermeable to potassium ions whereas the three open models are most probably permeable. We then assume the C-terminal domain can be approximated as a rigid body and measure its movement relative to the closed model of Kir1.1. Any rotation about the pore axis is measured by examining the movement of center of mass of the Cα atoms of the β-loop of one monomer (residues 329 to 339 incl, Kir1.1. numbering). We assessed the movement upwards towards the membrane of the C-terminal domain by examining the movement of the center of mass of all four G-loops (residues 302-305 incl.). These metrics show what we expect, i.e. that the C-terminal domains of all models except the closed model are closer to the membrane and the CTDs of the open models are twisted by 10-30° compared to the closed model.

1.3 Analysis of the closed, pre-open and open Kir1.1 models

The 47 residues that shift the $pH_{0.5}$ by more than 0.4 pH units were first mapped onto each of the five models. The proximity and interactions between all 188 (4×47) residues were then analysed. First all hydrogen bonds between the residues are calculated. We assumed a hydrogen bond to have formed if the distance between the donor and acceptor was less than 3.8 Å and the angle subtended by the donor, hydrogen and acceptor was less than 60°. Salt bridges were assumed to have formed if the sidechains of acidic and basic residues are separated by less than 3.8 Å. Finally, proximity between two residues was tested by first calculating the accessible surface area of each sidechain using a probe of varying radius (A and B) and then calculating the accessible surface area (ASA) of both sidechains together (C). If $(A+B) > C$ then we assume the two residues are packing against one another. The effect of varying the probe radius in the range 0.2-1.2 Å was examined and we decided on a probe radius of 1.0 Å by comparing the probe radius to the predicted thermal fluctuation (see below). This analysis was also done using VMD[8].

Each of the 188 residues was then assigned to a node in a graph and edges between residues were added where the above analysis indicated that a hydrogen bond or a salt bridge was present or when the two residues were in close proximity to one another. Only one edge between two residues was permitted and hence a salt bridge takes priority over a hydrogen bond which takes priority over an edge indicating proximity. Edges were added between

two proximal residues if difference in ASA is > 10 Å^2. This ensured that the edges indicating proximity are meaningful and that the resulting packing is likely to stabilise the protein. The clusters (connected subgraphs) of the resulting undirected graph were then identified and, for example, the distribution of cluster sizes and which residues are involved in each cluster were then recorded. This analysis was performed using the NetworkX python module[14]. It is common to measure the change in accessible surface area to water of a chemical moiety as means to estimate for the partitioning free energy[15]. Since the mutated sidechains were typically buried or exposed to lipid whether the channel is open or closed we are not using the accessible surface area in this way. Instead we are assessing how the packing between residues changes as the channel changes conformation and hence are assuming that the more tightly packed the interior of the channel, the lower its free energy. As discussed in the next section, in this context the radius of the probe is a simple way of including some measure of the thermal fluctuation inherent in the protein under the experimental conditions.

1.4 Relating the probe radius to the predicted atomic root mean square fluctuation

The Debye-Waller B-factors (B) measured by x-ray crystallography for each atom can be related to the average root mean square fluctuation (RMSF, $\langle \Delta R^2 \rangle^{1/2}$) via[16,17]

$$B = \frac{8\pi^2}{3} \langle \Delta R^2 \rangle .$$

The interpretation is often difficult since the B-factors also contain a component of the statistical error arising from inferring the position of the atom from the observed electron density. This contribution, however, can be minimised by examining high resolution crystal structures. None of the available structures of inwardly-rectifying potassium channels are of sufficiently high resolution, however, there are several high resolution structures of similar tetrameric cation channels, including KcsA[18] (PDB:1K4C, 2.0 Å resolution), NaK[19] (PDB:3OUF, 1.55 Å resolution) and MthK[20] (PDB:3LDC, 1.45 Å resolution). We calculated the estimated RMSF using the above approach for all the Cα carbons for each of these channels. Averages over the entire pore (80 residues) or just the selectivity filter (7 residues) are plotted in Figure S4. The RMSF is hence estimated to be in the range 0.7-0.8 Å in the filter and 0.9-1.0 Å for the pore. Using a probe radius in this range in the above proximity analysis is therefore a simple but effective method of modelling the thermal fluctuations in the protein. Indeed when we analysed the connectivity of the graphs in each model of Kir1.1 (Figure S5) we observed a sudden increase in the size of the largest sub-graph when the probe radius was in the range 0.8-1.0 Å. We therefore used a probe radius of 1.0 Å to interpret the connectivity of the graphs. Noskov et al.[17] also estimate that the RMSF of atoms in the filter of the KcsA channel to be roughly on the order of 1.0 Å.

2 Supplementary Tables

Table S1. (Refers to Figure 1) This is contained within a separate file and lists the mutations analysed and their effect on the measured $pH_{0.5}$ and Hill coefficients. If a simple alanine mutation led to a channel with no expression, a more conservative mutation was tried. If this also failed, in some cases a co-expression with wildtype was then attempted. Standard errors were calculated in the usual way and are also included.

Table S2. (Refers to Experimental Procedures and Figure 2 and Figure 3). This describes (i) which homologs were used for each model of Kir1.1 and (ii) a series of simple metrics (explained in the text above) which characterise how open the bundle crossing of each model is and how close the CTD is to the TMD.

3 Supplementary Movies

Movie S1. (Refers to Figure 1) The model of closed Kir1.1 is rotated showing which positions (a) were mutated (grey), (b) where mutation resulted in no functional activity (orange), (c) where mutation changed the $pH_{0.5}$ by less than 0.4 pH units (green), (d) where $pH_{0.5} < 6.0$ (blue) and (e) where $pH_{0.5} > 6.8$ (red, 47 positions).

Movie S2. (Refers to Figure 3) Five separate networks identified in the closed state fuse together forming a single network in the pre-open state which persists in the open state. The five largest networks (four identical in the TMD and one connecting all the G-loops in the CTD) in the closed state are shown on the model using space-filling spheres. As an aid to understanding, a morph from the closed to the pre-open state is shown, demonstrating how the conformational changes cause these networks to fuse together forming a single large network. This network remains approximately the same even when the channel undergoes further conformational changes and opens. This is illustrated by a morph from the pre-open to open state (Open-Kir3.2 model).

4 Supplementary Figures 1–6

All figures herein are referred to in either the main body of the paper or the **Supplementary Methods** section.

References

1. Tao, X., Avalos, J. L., Chen, J. & MacKinnon, R. Crystal structure of the eukaryotic strong inward-rectifier K+ channel Kir2.2 at 3.1 Angstrom resolution. *Science* **326**, 1668–74 (2009).
2. Hansen, S. B., Tao, X. & MacKinnon, R. Structural basis of PIP2 activation of the classical inward rectifier K+ channel Kir2.2. *Nature* **477**, 495–8 (2011).
3. Bavro, V. N., De Zorzi, R., Schmidt, M. R., Muniz, J. a. R. C., Zubcevic, L. *et al.* Structure of a KirBac potassium channel with an open bundle crossing indicates a mechanism of channel gating. *Nat Struct Mol Biol* **19**, 158–63 (2012).

Model	N-terminus	Slide & TM1 helices	EC loop	P-helix, Filter, TM2 helix & CTD	A180 $C\alpha$–$C\alpha$ distance (Å)	Pore radius (Å)	CTD (°)	CTD height (Å)
Closed	Kir2.2				15.8	1.5	–	–
Pre-open	Kir2.2 with PIP_2				14.8	2.1	2.1	5.3
Open-Kir3.2	Kirchim	Kir3.2	Kir2.2	Kir3.2	18.5	3.0	10.0	6.7
Open-KirBac-EM	KirBac1.1EM		Kir2.2	KirBac1.1EM	20.2	3.8	31.3	7.2

Table S2 (Refers to Experimental Procedures and Figure 2 and Figure 3) Construction of the Kir1.1 homology models. Also given are the pore radius, measured over a 7 Å window, and the angle and height of the CTD measured relative to the Closed model. The PDB codes for each homolog are given in the **Supplementary Methods** and an alignment can be found in Fig S6.

4. Whorton, M. R. & MacKinnon, R. Crystal Structure of the Mammalian GIRK2 K(+) Channel and Gating Regulation by G Proteins, PIP(2), and Sodium. *Cell* **147**, 199–208 (2011).

5. Domene, C., Doyle, D. A. & Vénien-Bryan, C. Modeling of an ion channel in its open conformation. *Biophys J* **89**, L01–3 (2005).

6. Nishida, M., Cadene, M., Chait, B. T. & MacKinnon, R. Crystal structure of a Kir3.1-prokaryotic Kir channel chimera. *EMBO J* **26**, 4005–15 (2007).

7. Sali, A. & Blundell, T. L. Comparative protein modelling by satisfaction of spatial restraints. *J Mol Biol* **234**, 779–815 (1993).

8. Humphrey, W., Dalke, A. & Schulten, K. VMD: visual molecular dynamics. *J Mol Graph* **14**, 33–8 (1996).

9. Lovell, S. C., Davis, I. W., Arendall, W. B., de Bakker, P. I. W., Word, J. M. *et al.* Structure validation by Calpha geometry: phi,psi and Cbeta deviation. *Proteins* **50**, 437–50 (2003).

10. Hess, B., Kutzner, C., van der Spoel, D. & Lindahl, E. GROMACS 4: Algorithms for Highly Efficient, Load-Balanced, and Scalable Molecular Simulation. *J Chem Theo Comp* **4**, 435–447 (2008).

11. Cuello, L. G., Jogini, V., Cortes, D. M., Pan, A. C., Gagnon, D. G. *et al.* Structural basis for the coupling between activation and inactivation gates in K+ channels. *Nature* **466**, 272–275 (2010).

12. Smart, O. S., Neduvelil, J. G., Wang, X., Wallace, B. A. & Sansom, M. S. P. HOLE: a program for the analysis of the pore dimensions of ion channel structural models. *J Mol Graph* **14**, 354–60 (1996).

13. Beckstein, O., Tai, K. & Sansom, M. S. P. Not ions alone: barriers to ion permeation in nanopores and channels. *J Am Chem Soc* **126**, 14694–5 (2004).

14. Hagberg, A. A., Schult, D. A. & Swart, P. J. Exploring Network Structure , Dynamics , and Function using NetworkX. In G. Varoquaux, T. Vaught & J. Millman, eds., *Proceedings of the 7th Python in Science Conference (SciPy2008)*, SciPy, pp. 11–15. Pasadena, CA USA (2008).

15. Richards, F. M. Areas, volumes, packing and protein structure. *Ann Rev Biophys* **6**, 151–76 (1977).

16. Halle, B. Flexibility and packing in proteins. *Proc Natl Acad Sci U S A* **99**, 1274–9 (2002).

17. Noskov, S. Y., Bernèche, S. & Roux, B. Control of ion selectivity in potassium channels by electrostatic and dynamic properties of carbonyl ligands. *Nature* **431**, 830–4 (2004).

18. Zhou, Y., Morais-Cabral, J. H., Kaufman, A. & MacKinnon, R. Chemistry of ion coordination and hydration revealed by a K+ channel-Fab complex at 2.0 A resolution. *Nature* **414**, 43–8 (2001).

19. Derebe, M. G., Zeng, W., Li, Y., Alam, A. & Jiang, Y. Structural studies of ion permeation and Ca2+ blockage of a bacterial channel mimicking the cyclic nucleotide-gated channel pore. *Proc Natl Acad Sci U S A* **108**, 592–597 (2011).

20. Ye, S., Li, Y. & Jiang, Y. Novel insights into K(+) selectivity from high-resolution structures of an open K(+) channel pore. *Nat Struct Mol Biol* **17**, 1019–23 (2010).

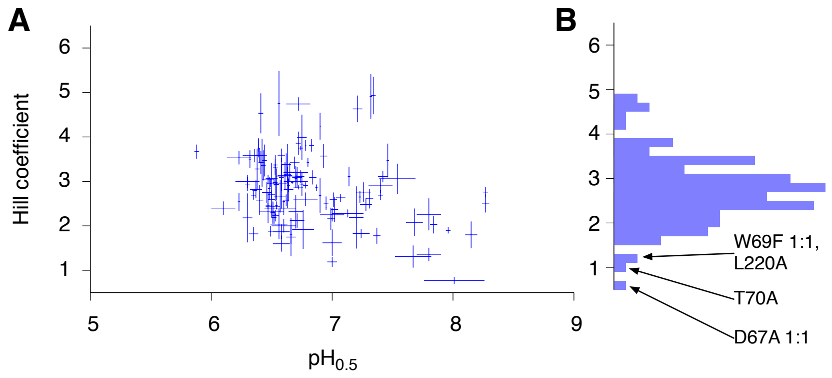

Figure S1 (Refers to Figure 1 and Figure 5) (**A**) Lack of correlation between the $pH_{0.5}$ and the Hill coefficients estimated from the electrophysiological data. Experimental estimates of the error in both quantities are plotted. (**B**) Unlike the $pH_{0.5}$ values, the estimated Hill coefficients form an approximately symmetric distribution with the wildtype (2.5 ± 0.2) being close to the center.

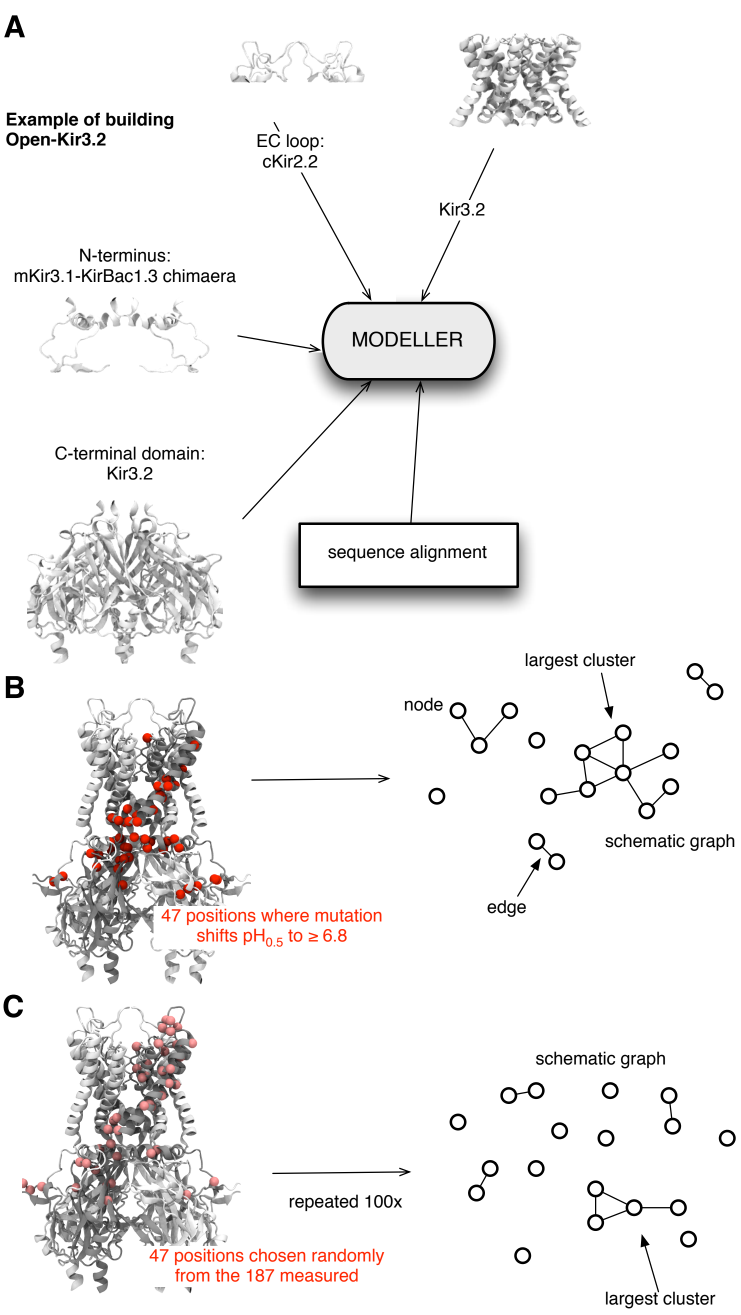

Figure S2 (Refers to Experimental Procedures and Figure 2) Schematic illustrating how homology models were built and analysed. (**A**) Although mainly built using one of the monomers from the R201A Kir3.2 PIP_2 bound structure[4], the Open-Kir3.2 model of Kir1.1 required several templates (see **Supplementary Table S2**) as shown. (**B**) The 47 positions which shift the $pH_{0.5}$ by more than 0.4 units are mapped onto each model. These define the nodes of a graph. Edges were added where residues either pack close to one another, are hydrogen bonded or form a salt bridge, as described in the **Supplementary Methods**. The connectivity of the resulting graph is then analysed, in particular the distribution of cluster sizes. (**C**) To check that these clusters did not arise by chance or simply because the open models are more compact, 47 positions were also selected at random from the 187 positions tested and the resulting graphs analysed. This was repeated 100 times for each model and the results compared to the set of experimentally identified residues (Figure S5).

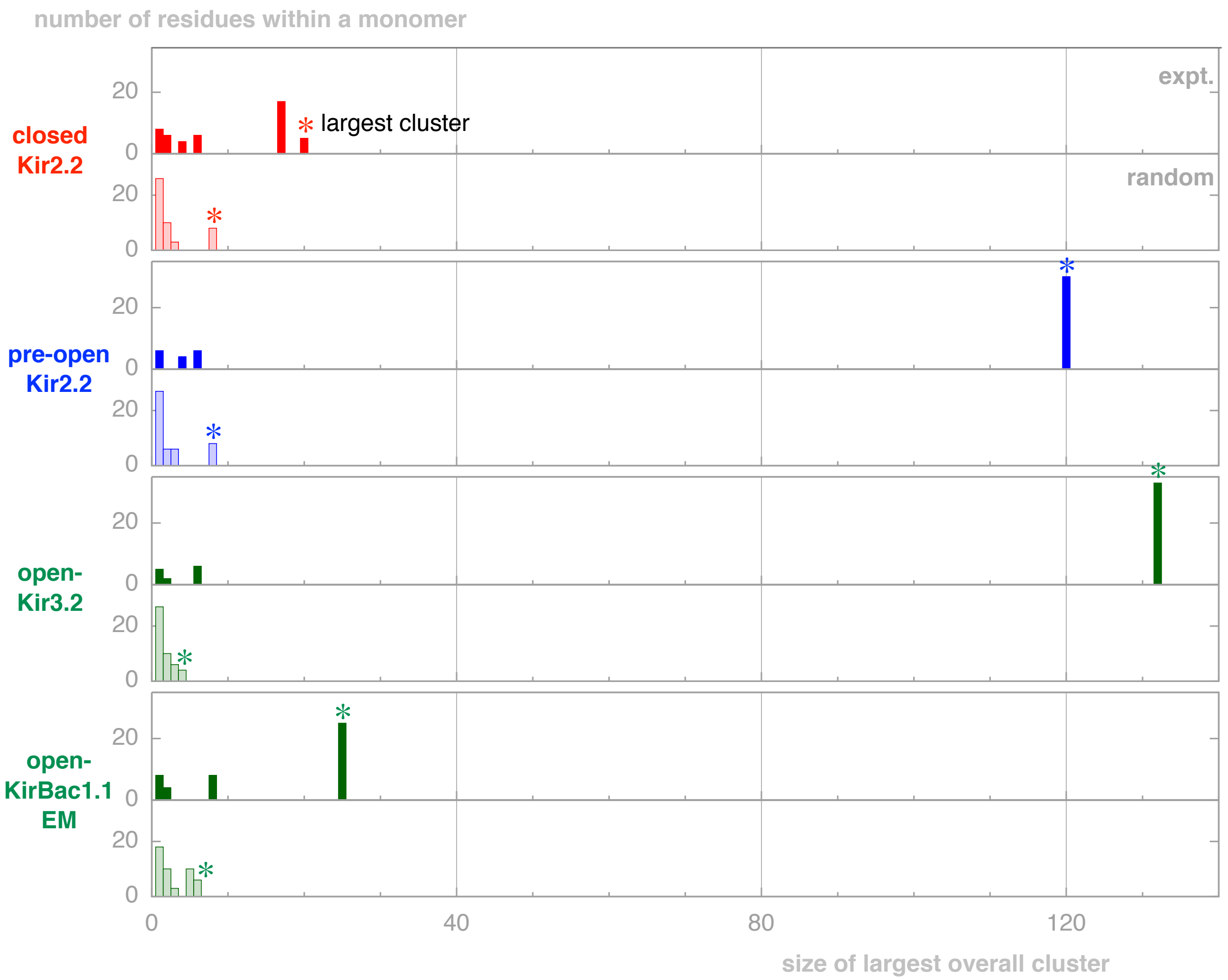

Figure S3 (Refers to Figure 2B) Large clusters form in the Pre-open and Open-Kir3.2 models but not in the Closed and control Open-KirBac-EM models. To test if these networks are significant the bottom panel for each model shows one example where 47 residues are chosen randomly and the resulting network analysed. This is repeated 100 times and the average and ranges are shown in Figure S5. The single largest cluster in each case is indicated by an arrow.

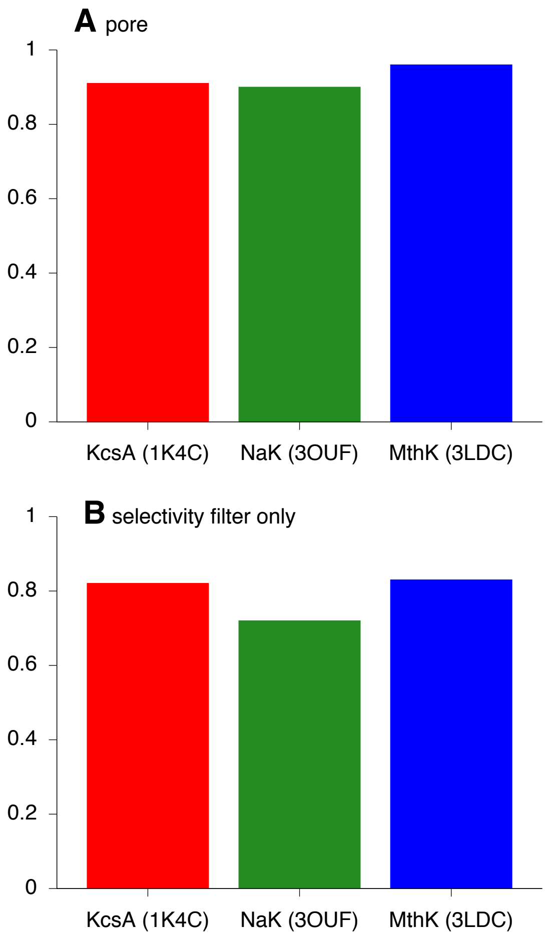

Figure S4 (Refers to Experimental Procedures and Figure 2B) The average RMSFs predicted from the Debye-Waller crystallographic *B*-factors for three high resolution ion channel structures are in the range (**A**) 0.9-1.0 Å for Cα in the pore and (**B**) 0.7-0.8 Å for Cα atoms in the selectivity filter. The structures are KcsA (PDB:1K4C, 2.0 Å resolution)[18], NaK (PDB:3OUF, 1.55 Å resolution)[19] and MthK (PDB:3LDC, 1.45 Å resolution)[20].

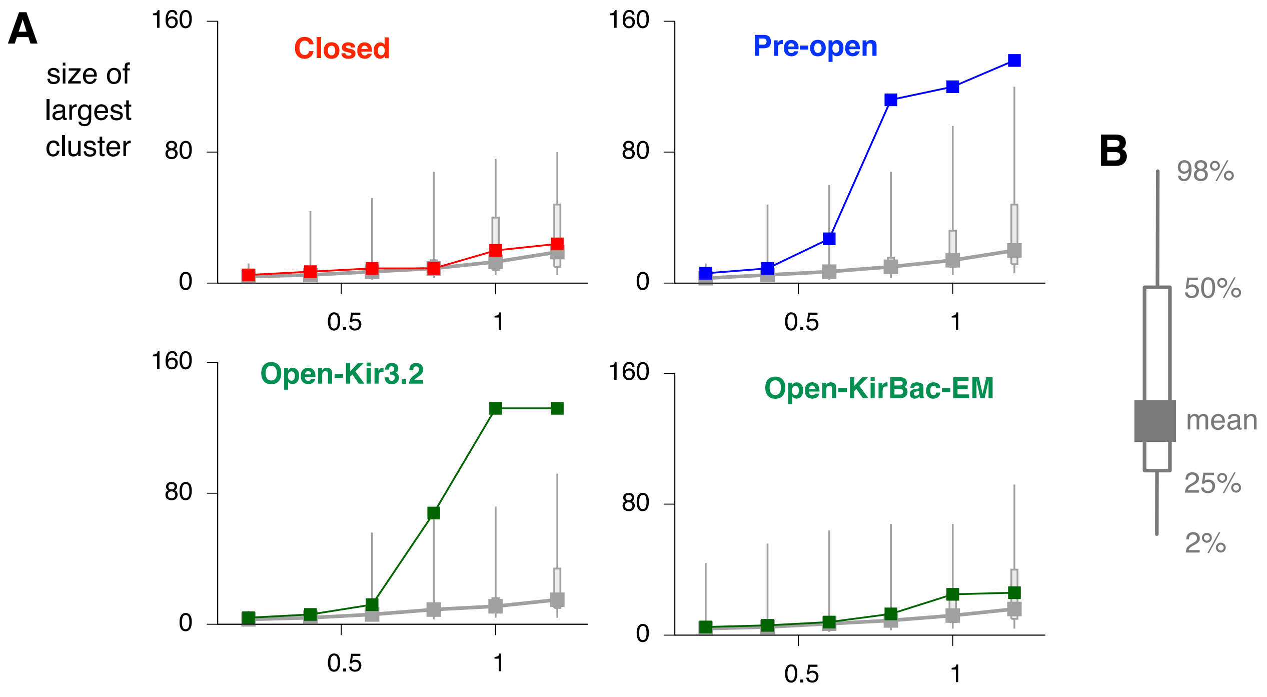

Figure S5 (Refers to Experimental Procedures and Figures 2C-F) The clustering observed when the experimentally identified 47 positions are mapped onto the pre-open and open state models of Kir1.1 is much greater than when graphs formed using sets of 47 randomly chosen positions are analysed. (**A**) How the size of the largest cluster varies with the probe radius. To test if the large clusters that form in the Pre-open and Open-Kir3.2 models are significant when the probe radius is > 0.6 Å we repeated the graph-based analysis for 100 models where 47 positions were randomly chosen from the 187 positions tested. This is repeated for all three models and the control Open-KirBac-EM model and yields a distribution of sizes for the largest cluster at each probe radius. This is drawn as a box-whiskers plot as defined in (**B**). At a probe radius of 1.0 Å the size of the largest experimental cluster is larger than any of the 100 randomly assigned cluster for the Pre-open and Open-Kir3.2 models but not for the closed or low-resolution Open-KirBac-EM models.

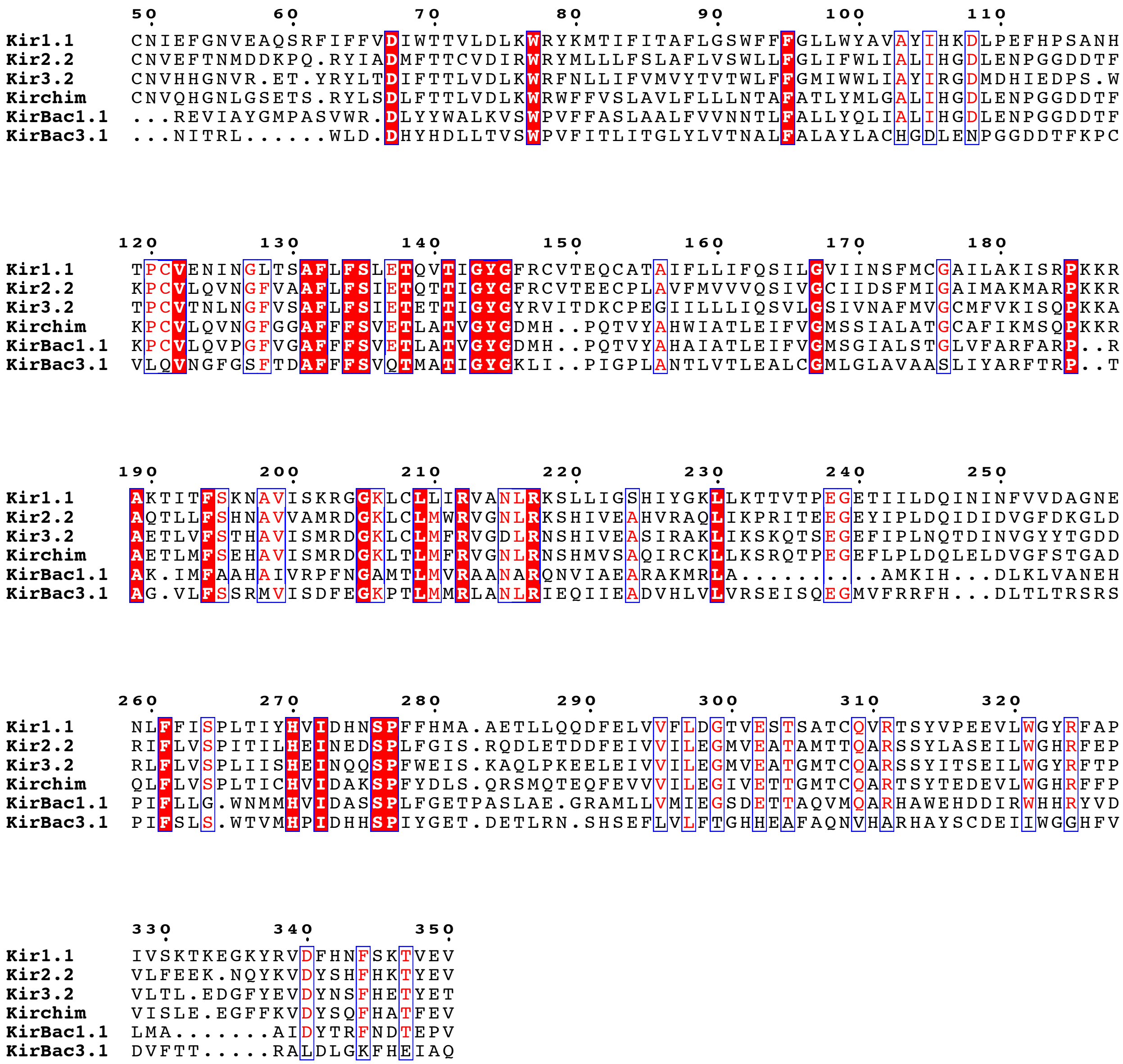

Figure S6 (Refers to Figure 3) There is a high degree of similarity between Kir1.1 and the homologs used to build the models of Kir1.1. The whole of Kir3.2 is not resolved in the crystal structure[4], in particular the N-terminus and the large extracellular loop. The former was modeled using the Kirchim structure and the latter Kir2.2. For more details about how the models were constructed see **Supplementary Table S2**
