# Supplementary figures and images for: State-Dependent Network Connectivity Determines Gating in a K+ Channel
Source: Structure. 2014 Jul 8;22(7):1037–46. doi: 10.1016/j.str.2014.04.018 (PMC4087272; doi:10.1016/j.str.2014.04.018)

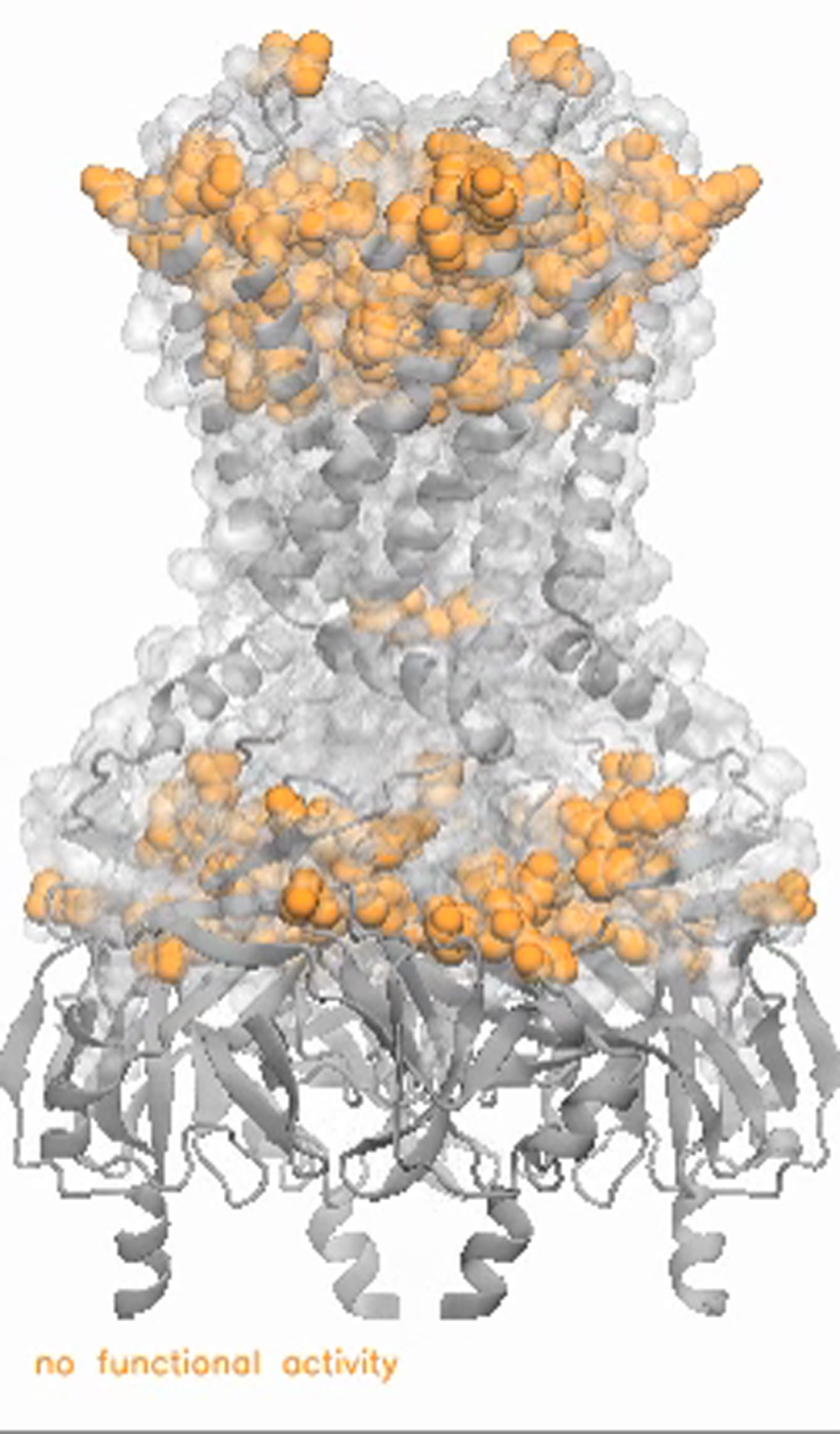

Supplement: Movie S1. The Model of Closed Kir1.1, Related to Figure 1 — The closed model of Kir1.1 is rotated, showing (A) which positions were mutated (grey), (B) where mutation resulted in no functional activity (orange), (C) where mutation changed the pH05 by less than 0.4 pH units (green), (D) where pH05 < 6.0 (blue), and (E) where pH05 > 6.8 (red, 47 positions). [file mmc3.jpg]

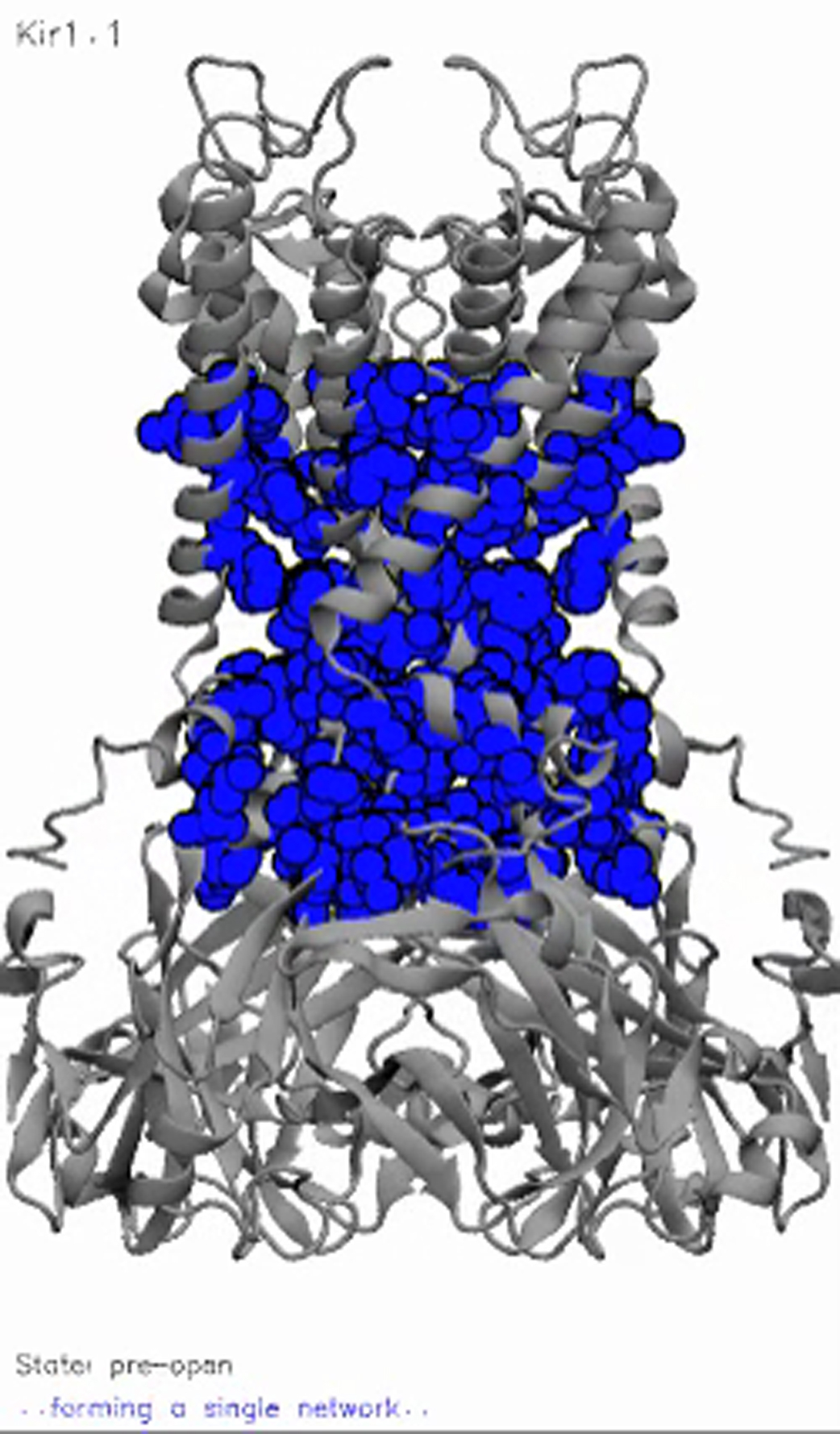

Supplement: Movie S2. Five Networks Identified in the Closed State Fuse Together, Forming a Single Network in the Pre-Open state, which Persists in the open State, Related to Figure 3 — The five largest networks (four identical in the TMD and one connecting all the G loops in the CTD) in the closed state are shown on the model using space-filling spheres. As an aid to understanding, a morph from the closed state to the pre-open state is shown, demonstrating how the conformational changes cause these networks to fuse together and form a single large network. This network remains approximately the same, even when the channel undergoes further conformational changes and opens. This is illustrated by a morph from the pre-open to open state (open-Kir3.2 model). [file mmc4.jpg]
